# Case report of metastatic prostate cancer masquerading as squamous cell carcinoma on the tip of the penis

**DOI:** 10.1016/j.eucr.2021.101804

**Published:** 2021-08-09

**Authors:** Nicholas W. Russo, Clifford E. Georges, Angelo A. Baccala

**Affiliations:** a University of South Florida Morsani College of Medicine, Lehigh Valley Campus, USA; b Lehigh Valley Health Network, Lehigh Valley Physician's Group, USA

**Keywords:** Penile metastasis, Prostate adenocarcinoma

## Abstract

We present a 76-year old man with a two year history of Gleason 9 prostate cancer (PCa) presenting with penile pain, erythema, and a fungating mass on the glans. Imaging at initial PCa diagnosis revealed confined disease. His prostate cancer was previously treated with radiation and androgen deprivation therapy (ADT) with initial laboratory response via prostate specific antigen (PSA) levels, however his PSA began to rise six months following cessation of ADT. Shared decision making resulted in partial penectomy for symptomatic management. Computed tomography (CT) and bone scan performed after surgery were not definitive for metastatic disease.

## Highlights

• Prostatic adenocarcinoma metastasis to the penis is a rare occurrence.
• Presentation can mimic that of squamous cell carcinoma.
• Treatment is often focused on improving quality of life.

## 1 Introduction

Metastasis of prostate cancer to the penis is extremely rare, making it difficult to establish prevalence. In addition, few cases are reported in the literature, with a poor prognosis of death within 6 months of diagnosis.^1^ The differential diagnosis includes primary penile malignancies such as squamous cell carcinoma (SCC), lymphoma, and sarcoma, metastatic spread from the pelvic and rectosigmoid region, and benign conditions such as Peyronie's disease. Clinical findings, including physical exam, and imaging will help elucidate etiology. The gold standard for diagnostic certainty remains the core needle biopsy. Treatment for metastatic spread of prostate cancer to the penis is patient dependent. Factors include patient condition, disease burden, and quality of life considerations.

## 2 Case presentation

A 76-year old man presented with a four month history of penile pain and erythema to the urology department. Symptom progression over the preceding months included burning with urination and difficulty retracting foreskin. Initial treatment with a two-week course of nystatin-triamcinolone cream was unsuccessful. Physical exam demonstrated a two-centimeter large fungating mass on the ventral surface of the distal shaft, which appeared to originate from the glans. Digital rectal exam revealed an enlarged, firm, and nodular prostate. The remainder of the physical exam was within normal limits. There was no palpable lymphadenopathy, no focal spinal tenderness, and a normal neurological exam without focal or gross motor weakness. Given the suspicious features of the mass for SCC, particularly the fungating and superficial nature, a discussion with the patient was made regarding management options including biopsy for definitive diagnosis versus partial penectomy. The patient opted for surgical treatment and tolerated surgery well. Pathology following treatment demonstrated Gleason 9 prostate cancer. CT scan of the pelvis was performed following penectomy to assess treatment response, and demonstrated a stable sclerotic lesion on the iliac bone with potential sclerosis inferior to this known lesion and no lymphadenopathy. Bone scan at this time demonstrated no definitive evidence of metastatic disease.

Past medical history is significant for Gleason 9 prostate adenocarcinoma diagnosed two years prior. Workup at the time included magnetic resonance imaging (MRI) of the prostate which demonstrated a Prostate Imaging Reporting & Data System (PI-RADS) 5 lesion, mildly enlarged pelvic lymph nodes, and a small sclerotic focus of the left iliac bone. Bone scan did not demonstrate evidence of osseous metastatic disease, and CT scan of the chest was normal. Given benign bone scan and following discussion with hematology oncology, it was believed the iliac lesion did not represent metastatic disease. An informed discussion was made with the patient involving treatment modalities, and the decision was ultimately made to pursue combination intensity-modulated radiation therapy and ADT with leuprolide acetate. The patient tolerated treatment well and demonstrated a decrease in PSA over the following two years. Following completion of ADT, PSA levels began increasing over the following year, prompting resumption of hormonal therapy. At this time the patient began experiencing his present penile pain and urinary symptoms.

## 3 Discussion

Metastatic cancer to the penis is a rare occurrence, making it difficult to establish prevalence. Cherian et al. published a review detailing site-specific etiologies of penile metastases, and found that 34 % of these cases originated from the prostate, with bladder cancer metastases as the next highest cause at 30 %.^2^ While the exact mechanism of metastatic spread is unknown and likely multifactorial, retrograde venous flow is the most commonly accepted etiology.^2^ A review of the published literature on penile metastasis from prostate cancer demonstrates an average age of 73 years old, with a primary cancer already diagnosed and no predilection for metastatic deposit to the root, shaft, or glans.^3^ Previous literature has reported that the most common presentation is malignant priapism,^3^ however genitourinary symptoms such as retention, hematuria, and pain are also potential presenting signs. The most common primary tumor of the penis is SCC.^4^ Clinical presentation will often demonstrate a painless skin nodule or ulceration, and given this readily visualized presentation it is often diagnosed early.^4^ Furthermore, because of the readily apparent clinical evidence, imaging is typically not required. Treatment for metastatic spread of prostate cancer to the penis is patient dependent. Factors include patient condition, disease burden, and quality of life. Generally, conservative management is indicated in situations prioritizing quality of life improvement. This includes nonmedical symptomatic management for patients with urethral outflow obstruction via cystotomy or suprapubic catheterization. For patients with prostate cancer metastasis to the penis, combination radiation and androgen deprivation or hormonal therapy can provide symptomatic relief.^5^ Radical penectomy is another viable treatment option, especially in instances of uncontrollable pain^4^. Of note, these patients often have metastatic deposits elsewhere in the body (e.g., bony metastases secondary to prostate cancer), and so surgical resection is often palliative rather than curative.

## 4 Conclusion

Penile metastases are rare, with poor prognosis. Squamous cell carcinoma is the most common malignancy of the penis, and should be on the differential. Biopsy is gold standard for diagnosis and helps delineate management. For prostate cancer, combination radiation and hormonal therapy and penectomy are viable treatment options. A values-based and informed approach with the patient is crucial to determining the most appropriate treatment modality, as management will often be aimed at improving quality of life.

## Funding

This research did not receive any specific grant from funding agencies in the public, commercial, or not-for-profit sectors.

## Declaration of competing interest

None.
